# Neural Substrates of Food Valuation and Its Relationship With BMI and Healthy Eating in Higher BMI Individuals

**DOI:** 10.3389/fnbeh.2020.578676

**Published:** 2020-12-04

**Authors:** Junaid S. Merchant, Danielle Cosme, Nicole R. Giuliani, Bryce Dirks, Elliot T. Berkman

**Affiliations:** ^1^Neuroscience and Cognitive Science Program (NACS), Department of Psychology, University of Maryland, College Park, MD, United States; ^2^Annenberg School for Communication, University of Pennsylvania, Philadelphia, PA, United States; ^3^Prevention Science Institute, Department of Special Education and Clinical Sciences, University of Oregon, Eugene, OR, United States; ^4^Department of Psychology, University of Miami, Coral Gables, FL, United States; ^5^Center for Translational Neuroscience, Department of Psychology, University of Oregon, Eugene, OR, United States

**Keywords:** subjective valuation, food-cue reactivity, healthy eating, BMI—body mass index, willingness-to-pay, fMRI

## Abstract

Considerable evidence points to a link between body mass index (BMI), eating behavior, and the brain's reward system. However, much of this research focuses on food cue reactivity without examining the subjective valuation process as a potential mechanism driving individual differences in BMI and eating behavior. The current pre-registered study (https://osf.io/n4c95/) examined the relationship between BMI, healthy eating, and subjective valuation of healthy and unhealthy foods in a community sample of individuals with higher BMI who intended to eat more healthily. Particularly, we examined: (1) alterations in neurocognitive measures of subjective valuation related to BMI and healthy eating; (2) differences in the neurocognitive valuation for healthy and unhealthy foods and their relation to BMI and healthy eating; (3) and whether we could conceptually replicate prior findings demonstrating differences in neural reactivity to palatable vs. plain foods. To this end, we scanned 105 participants with BMIs ranging from 23 to 42 using fMRI during a willingness-to-pay task that quantifies trial-by-trial valuation of 30 healthy and 30 unhealthy food items. We measured out of lab eating behavior via the Automated Self-Administered 24 H Dietary Assessment Tool, which allowed us to calculate a Healthy Eating Index (HEI). We found that our sample exhibited robust, positive linear relationships between self-reported value and neural responses in regions previously implicated in studies of subjective value, suggesting an intact valuation system. However, we found no relationship between valuation and BMI nor HEI, with Bayes Factor indicating moderate evidence for a null relationship. Separating the food types revealed that healthy eating, as measured by the HEI, was inversely related to subjective valuation of unhealthy foods. Imaging data further revealed a stronger linkage between valuation of healthy (compared to unhealthy) foods and corresponding response in the ventromedial prefrontal cortex (vmPFC), and that the interaction between healthy and unhealthy food valuation in this region is related to HEI. Finally, our results did not replicate reactivity differences demonstrated in prior work, likely due to differences in the mapping between food healthiness and palatability. Together, our findings point to disruptions in the valuation of unhealthy foods in the vmPFC as a potential mechanism influencing healthy eating.

## Introduction

Global obesity rates have increased nearly 3-fold since 1975 (Abarca-Gómez et al., 2017), and current estimates indicate that middle-aged adults have the highest prevalence of obesity (Hales et al., 2020). Body mass index (BMI) during the early middle-age years (i.e., ages 35–45; Medley, 1980) predicts life expectancy (Peeters et al., 2003), and research suggests that prolonged life-style changes during this period have the ability to reverse the deleterious effects of poor health (Howden et al., 2018). A confluence of factors has been examined to understand the development of overweight and obesity status during early middle-age adulthood, but one factor that has received relatively little attention is the change in neural circuitry underlying decision-making during this stage of life (Samanez-Larkin and Knutson, 2015). In particular, recent work points to the promise of examining subjective valuation as an underlying process involved in dietary choice and changes in eating behavior that contribute to higher BMI (Rangel, 2013; Giuliani et al., 2018).

Subjective valuation is the neurocognitive process by which an individual assigns value to an item, which is used to guide decision making (Rangel, 2013; Berkman et al., 2017a). When applied to food-based decision making, this framework allows for the integration of motivational and self-relevant factors contributing to the subjective valuation process when making choices about healthy and unhealthy foods (Rangel, 2013; Berkman et al., 2017b; Berkman, 2018). Despite the promise of the value-based approach to understanding the neurocognitive mechanisms contributing to weight and eating behavior, much of the health neuroscience research has focused on the link between food-cue reactivity in the brain, and its relation to eating behavior and BMI (Tetley et al., 2009; Yokum et al., 2011; Gunes et al., 2012; Murdaugh et al., 2012; Verdejo-Román et al., 2017; Harding et al., 2018; Contreras-Rodriguez et al., 2020). Thus, the current pre-registered study (https://osf.io/n4c95/) aims to expand health neuroscience research in this area by examining the relationship between BMI, healthy eating, and the brain regions involved in subjective valuation of healthy and unhealthy foods in a sample of early middle-aged adults with higher BMI.

### Food-Cue Reactivity, Self-Regulation, and the Relationship With BMI and Eating Behavior

Much of our understanding of the relationship between higher BMI, eating behavior, and the brain's reward system has been examined through the lens of the “food addiction” model (Smith and Robbins, 2013). The reward system is comprised of dopamine producing neurons in the ventral tegmental area (VTA) that project to the nucleus accumbens (NAcc) and prefrontal cortex, which are involved in motivation and pleasure, but show dysregulated activity in individuals with higher BMI similar to what has been observed in individuals with substance addictions (Volkow et al., 2013a). The food addiction model proposes that the heightened reactivity to food cues in individuals with higher BMI leads to an inability to regulate food intake, thereby leading to overeating and weight gain (Volkow et al., 2013b). Neuroimaging research has confirmed heightened reactivity to food-cues in the brain's reward system, as well as brain regions associated with attention and emotional salience in relation to BMI and eating behavior (Yokum et al., 2011; Smeets et al., 2012; Verdejo-Román et al., 2017). For instance, Lawrence and colleagues (2012) demonstrated that food-cue reactivity in the NAcc predicts the amount of subsequent food consumption, and interactions between NAcc reactivity and self-reported self-control predicted BMI (Lawrence et al., 2012). Moreover, this work has shown that the relationship between food-cue reactivity and eating behavior and weight is specific to desirable foods (e.g., high-calorie foods; Yokum et al., 2011; Stice and Yokum, 2018; Yokum and Stice, 2019; Verdejo-Román et al., 2017), and can prospectively predict later consummatory behavior and outcomes in weight-loss programs (Demos et al., 2012; Murdaugh et al., 2012; Kroemer et al., 2016). Together, these findings suggest that biases in the subjective valuation system, particularly for unhealthy, desirable foods might be the process linking brain reactivity, BMI, and eating behavior.

Self-regulation research provides a complementary framework for health neuroscience research by providing neurocognitive mechanisms leading to healthier behavior. In the context of eating behavior, self-regulation involves the reduction of food cravings through cognitive regulation strategies that engage executive control regions of the brain, which is thought to break the link between food-cue reactivity and eating behavior (Giuliani et al., 2018). For instance, the process of reappraising personally craved foods to make them a less desirable elicited relatively greater activity in the dorsolateral prefrontal cortex (dlPFC) and dorsal anterior cingulate cortex (dACC), and this activity was negatively correlated with BMI in a sample of young adults (Giuliani et al., 2014). Importantly, self-regulation, with respect to eating, can be trained (Boswell and Kober, 2016), and greater self-regulatory ability protects against developing obesity (Duckworth, 2011). Indeed, recent studies using this framework have targeted the brain's self-regulation system using real-time neurofeedback, and demonstrated measurable reductions in palatability ratings and consumption of unhealthy foods after neurofeedback training (Spetter et al., 2017; Kohl et al., 2019). For purposes of the current study, healthy eating goals are another top-down factor integrated during valuation. If health goals are active during valuation, they may be indexed by activation in cognitive control brain regions (Tusche and Hutcherson, 2018).

### Subjective Valuation as a Mechanism Driving BMI and Eating

Subjective valuation is the neurocognitive process wherein an individual assigns value to items in order to help guide decision-making, and can be measured as the correspondence between self-reported value and level of activity in brain regions commonly associated with reward processing (Bartra et al., 2013; Berkman et al., 2017a). Examining weight and eating behavior within this framework provides a means to incorporate findings from the food-cue reactivity and self-regulation research, and better operationalizes the neurocognitive mechanism involved in food-based decision making as it relates to BMI and eating behavior (Giuliani et al., 2018). Particularly, a value-based approach posits that the brain's reward, salience, and self-regulation networks comprise general-purpose value systems that assign subjective value during decision making, and suggests a special role for the ventromedial prefrontal cortex (vmPFC) in integrating disparate value signals (Chib et al., 2009; Hare et al., 2011; Berkman, 2018). This neurocognitive mechanism has been supported by a meta-analysis of over 200 studies that established a highly reliable, positive relationship between subjective value (i.e., how much one is willing to pay for an object, or “willingness-to-pay”) and the blood-oxygenation-level-dependent (BOLD) response in the brain's reward centers, particularly the vmPFC and anterior ventral striatum (aVS; Bartra et al., 2013). Within this framework, multiple factors, such as healthy eating goals and food desirability, are integrated into a common value signal that guides choices (Berkman et al., 2017b; Berkman, 2018). For example, research has demonstrated that the value signal in the vmPFC is modulated by activity in the dlPFC that responds to immediate task demands, such as focusing on food healthiness, which in turn drives the selection of food-based decisions (Hare et al., 2011; Hutcherson et al., 2012).

To date, few studies have utilized willingness-to-pay paradigms, in which subjective values of foods are measured directly via bids in a food auction, to examine the relationship between eating behavior, BMI, and brain responses to the food cues (Verdejo-Román et al., 2017; Contreras-Rodriguez et al., 2020). The two studies that have examined the relationship between willingness-to-pay and BMI did not model the linear relationship between BOLD response and bid amount for the food items, but instead examined differences in the overall brain activation for different food categories. Thus, the previous approach is more akin to food-cue reactivity paradigms, which compare average activation to discrete categories of stimuli, rather than to the approach used in subjective valuation paradigms, because the continuously-rated self-reported values (i.e., bids) were disregarded.

### Current Study

The current pre-registered analyses aim to extend our understanding of the relationship between the neurocognitive process of subjective valuation, eating behavior, and BMI by utilizing data collected for a larger intervention study. The project was pre-registered after data were collected, but before any of the registered analyses were conducted (https://osf.io/n4c95/). The original study targeted adults who were at-risk for developing weight-related health consequences—a community sample of early middle-aged adults (i.e., 35–45 years old) with higher BMI (i.e., BMI = 25–40) who had explicit healthy eating goals, but were not actively enrolled in any dietary intervention during the time of testing. Recent estimates indicate that middle-aged adults have the highest prevalence of obesity (Esteban et al., 2019), but research suggests that life-style changes during the early middle-age years have the ability to reverse the negative impacts of poor health (Howden et al., 2018). The current analyses were conducted on data collected at the baseline session before any intervention took place. To elucidate brain regions supporting valuation, participants completed the willingness-to-pay paradigm—which provides an explicit measure of self-reported value for different food types—while in the MRI scanner.

First, we examined the functioning of the valuation system using neuroimaging, and its relationship to BMI and healthy eating. That is, we assessed the degree to which our sample showed the expected relationship between self-reported value and BOLD response in the brain's valuation system during food choices, and whether valuation was related to BMI and healthy food consumption. Due to the relative novelty of this analysis, we did not have *a priori* hypotheses about the existence or direction of this relationship. However, if vmPFC activity tracked value *and* was related to BMI or healthy eating, we could infer that the valuation process may drive some of the individual differences for these measures. Second, we compared the valuation of healthy and unhealthy foods separately, and how they related to BMI and healthy eating. Specifically, we compared the coupling of self-reported value and BOLD response between decisions made for healthy vs. unhealthy food items, and examined if this predicted BMI and/or healthy eating. Based on studies of food-cue reactivity that suggest an overreactivity bias to unhealthy foods in higher BMI individuals (Yokum et al., 2011; Verdejo-Román et al., 2017), we expected to see differences in the valuation process between food categories, but did not have strong predictions as to the nature or direction of the relationship with BMI and healthy eating. Finally, we aimed to provide a conceptual replication of Verdejo-Román et al. (2017) who also used the willingness-to-pay paradigm, but disregarded self-reported value. Specifically, we examined if activation differences between healthy and unhealthy foods (i.e., ignoring self-reported value) showed a similar pattern of results as when Verdejo-Roman and colleagues contrasted unpalatable and palatable foods.

## Methods

### Participants

A sample of 105 higher BMI, middle-aged individuals who had explicit goals to eat more healthily were screened and enrolled into a larger, longitudinal project investigating the neural predictors of dietary change. Inclusion criteria for the overarching study included (1) overweight or obese status as defined by the Center for Disease Control and Prevention (i.e., approximate BMI between 25 and 40); (2) early, middle-age (i.e., 33–45 years old); (3) no psychiatric, neurological, or eating disorders; (4) no fMRI contraindications; (5) not actively enrolled in a diet program or any other type of eating intervention; and (6) self-reported desire to eat more healthily. The current analysis included all participants that completed baseline measures of eating behavior, BMI and usable fMRI data during the willingness-to-pay experiment. Ten participants were excluded for non-compliance, not showing up, or the decision to drop out before completion of the baseline session. Two participants were excluded because of technical error during the willingness-to-pay task, and one participant was excluded from analyses of healthy eating because of technical error. A final sample of 93 participants (77 female, mean (SD) age = 39.25 (3.50); mean (SD) BMI = 31.41 (3.91) were included in all analyses, except for analyses of healthy eating, which had a sample 92 participants. Results from this sample were reported in a separate paper comparing alternative neurocognitive models of self-control using trial-level data (Cosme et al., 2019). However, the current analyses are unique in that they relate the valuation process to real world measures of health (i.e., BMI and eating behavior) using participant-level data. Importantly, the analytic plan in our pre-registration was not based on results from the previous analyses, and what was known about the data at the time of registering was included in the pre-registration document for complete transparency.

### Protocol and Measures

Participants were recruited from the community through a combination of online, newspaper, and public advertising. Interested participants were screened for exclusion criteria via phone, and eligible participants were sent a package of materials to complete before arriving for their baseline session. Participants were scheduled for their baseline lab visit, and asked to complete the package of measures within 24 h of the visit. Pre-baseline measures included demographic information, assessments of eating behavior, and a battery of psychometric measures assessing self-control, impulsivity, and other relevant affective and motivational factors (see https://osf.io/n4c95/ for full list of measures). Participants were instructed to not eat anything for at least an hour before the baseline visit. During the baseline visit, participants were consented, screened for MRI contraindications, and instructed on the requirements of the study. Weight and height were measured using a commercially available weight scale and wall ruler, and BMI was calculated by dividing the participant's weight in kilograms by squared height in meters. Eating behavior was assessed via the Automated Self-Administered 24-Hour (ASA24®) Dietary Assessment Tool (Subar et al., 2012) which allows for the calculation of the Healthy Eating Index (HEI; Guenther et al., 2013) using equations provided by the developers of the ASA (https://epi.grants.cancer.gov/asa24/resources/hei.html). The HEI quantifies healthy eating behavior based on the dietary guidelines for Americans. Following the recommendations proposed by the developers of this measure, participants completed the ASA within a few days prior to and during the baseline visit to obtain a more representative estimate of daily eating behavior.

**Figure 1:**
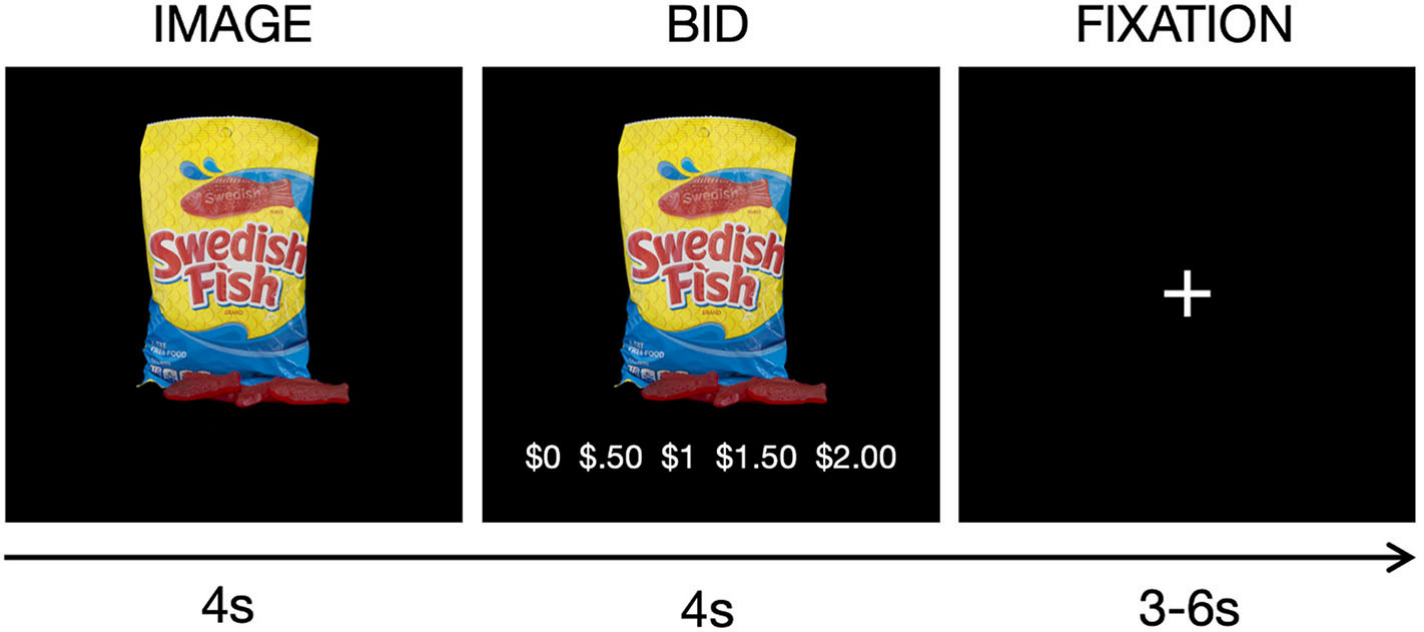
Task schematic for the willingness-to-pay task. On each trial, a snack food was presented for 4 s, followed by a 4 s bid period, and jittered fixation period between trials (M = 4.38 s). Snack foods were healthy (e.g., fruit) or unhealthy (e.g., candy) items.

The current study examined performance during the scanner-compatible willingness-to-pay task that quantifies trial-by-trial valuation of 30 healthy and 30 unhealthy snack food items, the healthiness of which was determined based on caloric density (Hutcherson et al., 2012), thereby providing both the self-report and BOLD responses used to index subjective valuation. Participants were given $2 prior to scanning for the purpose of bidding, and told that they would be bidding on snack food items during the scan for a chance to win one of the snacks. For each item, they were required to make bids on a scale from $0 to $2 dollars in increments of $0.50 based on how willing they are to pay to obtain the snack (Figure 1). To ensure truthful bids, we employed the rules of a Becker-DeGroot-Marschak auction (Becker et al., 1964; Plassmann et al., 2007). Briefly, they were instructed to treat each trial as if they had a fresh $2, and told that one of the snack items would be chosen at random at the end of the scan as the actual auction item they may win. If the bid they made for the selected item was higher than a randomly generated bid amount, they won the snack and were given the remainder of the $2 if the bid was below $2. If their bid was equal to or lower than the randomly generated bid, they did not win the snack item, and were refunded the full $2. All food stimuli were presented on black backgrounds, and the food item was centered such that the food stimulus took up most of the image space without crossing the margins. The task was presented using the PsychToolBox package for MATLAB (Brainard, 1997), and responses were made using a five-button button-box provided by the Lewis Center for Neuroimaging at the University of Oregon.

### MRI Data and Processing

Neuroimaging data were acquired on a 3T Siemens Skyra scanner at the University of Oregon Lewis Center for Neuroimaging. We acquired a high-resolution anatomical T1-weighted MP-RAGE scan (TR/TE = 2500.00/3.43 ms, 256 × 256 matrix, 1 mm thick, 176 sagittal slices, FOV = 208 × 208 mm), functional images with a T2^*^–weighted echo-planar sequence (72 axial slices, TR/TE = 2000.00/27.00 ms, 90-degree flip angle, 100 × 100 matrix, 2 mm thick, FOV = 208 × 208 mm, multiband acceleration factor = 3), and opposite phase encoded echo-planar images to correct for magnetic field inhomogeneities (72 axial slices, TR/TE = 6390.00/47.80 ms, 90-degree flip angle, 104 × 104 matrix, 2 mm thick, FOV = 208 × 208 mm). The willingness-to-pay task was acquired over 357 functional volumes scanned as one run.

Neuroimaging data were preprocessed using fMRIPrep 1.1.4 (Esteban et al., 2019). Briefly, anatomical images were segmented and normalized to MNI space; functional images were skull-stripped, susceptibility distortion corrected, realigned, slice-time corrected, co-registered and warped to the normalized anatomical image (see https://osf.io/n4c95/ for full report of the preprocessing pipeline). Normalized functional data were then smoothed (6 mm FWHM) in SPM12 (http://www.fil.ion.ucl.ac.uk/spm). Subject-level, voxel-wise multiple linear regression was calculated using AFNI's 3dREMLfit (Cox, 1996), using functions representing each condition convolved with a standard hemodynamic response function, each condition convolved with a standard hemodynamic response function with the amplitude modulated by trial-wise bid amount (in the case of research questions 1 and 2), motion regressors, and a “trash” regressor indicating images with motion artifacts (e.g., striping) identified via automated motion assessment (Cosme et al., 2018). For all group-level, whole-brain analyses, the cluster correction value was calculated automatically by using the -Clustsim option in AFNI's 3dttest++, which sends the input volumes directly to the 3dClustSim program after simulating the noise volumes by randomizing and permuting the input datasets. This model-free approach is more accurate at controlling the false positive rate compared to simulating noise using a mixed-model estimation of the autocorrelation function. This yielded a voxel-wise threshold of *p* < 0.001 and cluster extent of k = 119 to achieve a whole-brain familywise error rate of α = 0.05 when nearest neighbor cluster definitions are set to 3 (i.e., voxel faces, edges, and corners touching count as part of a contiguous cluster). Despite using the 3dttest++ program to determine cluster thresholds, we used AFNI's 3dMEMA program for group-level statistics because it better accounts for subject-level variance. All code used for neuroimaging analysis available (https://github.com/UOSAN/CHIVES_WTP_scripts).

### Overall Valuation

To address our first research question regarding neural function during the valuation process and its relation to BMI and healthy eating, we conducted a series of ROI and whole-brain analyses. First, we calculated subject-level whole-brain maps comprised of voxel-wise beta values that quantify the linear relationship between bid value for each food item and the corresponding BOLD response during the decision making period. Because prior research has established a set of brain regions comprising the valuation system, we first explored the valuation process within *a priori* regions of interest (ROIs) obtained from the Bartra et al. (2013) meta-analysis that best mapped on to our task parameters. Specifically, we used the vmPFC and anterior ventral striatum (aVS) ROIs from Figure 9 (Bartra et al., 2013, page 423) that reliably demonstrated a positive relationship with subjective value for primary and monetary rewards, and the anterior cingulate cortex (ACC) and bilateral anterior insulae (AI) from **Figure 3C** (Bartra et al., 2013, page 417) that showed a conjunction between positive and negative relationships with subjective value (available here: https://www.sas.upenn.edu/~mcguirej/meta-analysis.html).

Within each ROI, we averaged the voxel-wise parameter estimates from each participant, and used a one-sample *t*-test against zero to assess if our sample showed evidence of neurocognitive valuation. We then used the parameter estimates from the vmPFC and aVS ROIs to assess their relationship with BMI using linear regression, and similarly regressed HEI on the same parameter estimates in a separate linear regression model. We focused on the vmPFC and aVS because of the reliability with which they have been implicated in prior studies of subjective valuation (Bartra et al., 2013). Lastly, we conducted whole-brain searches to confirm the results of the ROI analysis, and to elucidate unpredicted brain regions involved in the subjective valuation process. The whole-brain search of subjective valuation was conducted by entering subject-level beta- and *t*-maps into AFNI's 3dMEMA program, which calculates a group-level mixed effects meta-analysis map that accounts for subject-level variance. Separate whole-brain searches for clusters relating valuation to BMI and HEI were calculated similarly in two separate analyses, but with the added covariates of interest (i.e., BMI and HEI, respectively).

### Valuation by Food Type

To address the second research question regarding differences in the valuation process between healthy and unhealthy foods, we conducted a series of behavioral and neuroimaging analyses. First, we assessed if self-reported value for healthy and unhealthy foods, or the interaction between the two, was related to BMI and healthy eating. To this end, we regressed BMI on subject-averaged bid amounts for healthy and unhealthy foods as separate independent variables in the first step of a linear regression model, and the interaction term between the two in the second step. This procedure was then repeated with HEI in a separate step-wise, linear regression model.

To compare the neurocognitive valuation between food types, we calculated a new set of subject-level models that separated healthy and unhealthy foods, and generated separate whole-brain maps for each food category comprised of voxel-wise beta values that quantified the linear relationship between bid amount and BOLD response. We then contrasted these maps (i.e., neurocognitive valuation for healthy > unhealthy foods) within subjects to evaluate differences in valuation across food categories across our sample. To examine differences in valuation for the food category within the vmPFC and aVS ROIs, we averaged the voxel-wise contrast value within each, and used a one-sample *t*-test against zero to examine if the coupling of self-reported value and corresponding BOLD amount was greater for one food category.

To assess the relationship between the neurocognitive valuation of healthy and unhealthy foods with individual differences in BMI and healthy eating, we calculated a set of regression models similar to the behavioral regression models above. We first combined the ROIs by averaging the parameter estimates across the vmPFC and aVS within subjects to use as our measure of neurocognitive valuation for healthy and unhealthy foods. We then regressed BMI on our neurocognitive measures of healthy and unhealthy foods as separate independent variables in the first step of linear regression model, and the interaction term between the two as in the second step. This procedure was repeated for HEI in a separate step-wise, linear regression model. Lastly, we conducted a set of whole-brain searches to confirm the results of our ROI analyses, and elucidate unpredicted brain clusters distinguishing the valuation for the food categories. Whole-brain search for differences in the neurocognitive valuation of healthy and unhealthy foods was calculated by entering subject-level contrast beta- and *t*-maps into AFNI's 3dMEMA program. Separate whole-brain searches for clusters relating this difference in valuation to BMI and HEI were calculated similarly in two separate analyses, but with the added covariates of interest (i.e., BMI and HEI, respectively).

### Replication Analysis

Finally, we attempted to conceptually replicate the findings from Verdejo-Román et al. (2017). In their paper, they similarly used the willingness-to-pay task, but categorized the foods into palatable (e.g., chocolate) and unpalatable (e.g., plain yogurt) conditions based on previously obtained ratings of palatability. Instead of modeling the relationship between bid amount and BOLD response, they contrasted overall activations for food categories (i.e., palatable > unpalatable) and found greater activation in “[bilateral] dorsal caudate, nucleus accumbens, ventral putamen, ventral tegmental area, intraparietal, ventromedial and dorsolateral prefrontal and anterior cingulate cortices, and the anterior insula extending to the lateral orbitofrontal gyrus” (p. 671; Verdejo-Román et al., 2017). Further, they demonstrated greater activation in striatal regions for obese compared to overweight individuals when comparing this contrast between groups.

Toward the goal of a conceptual replication, we examined if our unhealthy > healthy reactivity contrast showed a similar pattern of activation to their palatable > unpalatable contrast, as well as the between group differences they report. To this end, we calculated a third set of subject-level models that ignored bid values, and examined the reactivity to healthy and unhealthy foods. In particular, we generated separate whole-brain maps for each food category comprised of voxel-wise beta values that quantified the amplitude of the BOLD response, disregarding its relationship with self-reported value. We contrasted these maps at the subject level, and entered the resulting contrast beta- and *t*-maps into AFNI's 3dMEMA program for a whole-brain search of activation differences between food categories. Lastly, we split our sample into overweight (BMI < 30; *N* = 37) and obese groups (BMI > 30; *N* = 56) in an attempt to replicate Verdejo-Román and colleagues (2017) between group contrast (obese > overweight, for the unhealthy > healthy contrast), and entered the corresponding beta- and *t*-maps into AFNI's 3dMEMA program for a between group analysis.

### Deviations From the Pre-registration

We conducted exploratory, follow up analyses that were intended to clarify the pattern of results of any significant results as needed, none of which were pre-registered. Also, when possible, we assessed the evidence in favor of the null hypothesis by calculating Bayes Factor for non-significant findings. We calculated Bayes Factor (BF_10_) as the ratio of the likelihood of the alternative hypothesis to the likelihood of the null hypothesis, and followed Lee and Wagenmakers (2014) heuristics for interpreting this value as the strength of evidence in favor of the null hypothesis: 1–1/3 = anecdotal evidence, 1/3–1/10 = moderate evidence, 1/10–1/30 = strong evidence, 1/30> = very strong evidence. Given that these relationships had not been previously explored, BF_10_ was calculated to clarify the nature of null results, and provide a possible explanation as to why such findings have not been reported in prior work.

## Results

### Overall Valuation

#### ROI Analyses

Each of the ROIs showed a positive, linear relationship between self-reported value and corresponding BOLD response, even after Bonferroni correction, suggesting that our sample displayed normative functioning of the valuation system: vmPFC (M = 5.93, SD = 7.70, 95% CI [4.34, 7.51]), *t*(92) = 7.42, *p* < 0.001; aVS (M = 4.56, SD = 5.53, 95% CI [3.42, 5.70]), *t*(92) = 7.95, *p* < 0.001; ACC (M = 4.74, SD = 8.49, 95% CI [2.99, 6.49]), *t*(92) = 5.38, *p* < 0.001; Left AI (M = 2.71, SD = 6.43, 95% CI [1.38, 4.03]), *t*(92) = 4.06, *p* < 0.001; Right AI (M = 2.32, SD = 5.47, 95% CI [1.19, 3.44]), *t*(92) = 4.08, *p* < 0.001. However, when regressing BMI and HEI on the parameter estimates from the vmPFC and aVS ROIs, we did not find a significant model fit, nor were any of the individual partial regression coefficients significantly related to BMI or HEI, even when controlling for hunger level and time since the last meal (all *p* > 0.14; Table 1A). Bayes Factor for each model overall indicated moderate evidence in favor of the null hypothesis, BF_10_ = 0.15 and.18 for BMI and HEI, respectively, suggesting that BMI and HEI may not be related to the functioning of the valuation system when making decisions about foods in general.

**Table 1:** Regression tables for **(A)** the relationship between overall neurocognitive valuation in the vmPFC and aVS with BMI and HEI, respectively; **(B)** the relationship between self-reported bids for healthy foods, unhealthy foods, and their interaction with BMI and HEI, respectively; **(C)** the relationship between neurocognitive valuation in the vmPFC and aVS combined parameter estimates for healthy foods, unhealthy foods, and their interaction with BMI and HEI, respectively.

**Model**	**Variable**	**B**	**SE**	**95% CI**	**β**	***t***	***p***
**A) RELATIONSHIP WITH OVERALL NEUROCOGNITIVE FOOD VALUATION**							
***BMI = vmPFC + aVS***							
0	Intercept	30.989	0.554	29.89, 32.09		55.958	<0.001
	vmPFC	0.073	0.061	−0.05, 0.19	0.143	1.199	0.234
	aVS	−0.002	0.084	−0.17, 0.17	−0.003	−0.028	0.978
Model summary: *F*_(2, 90)_ = 0.92, *p* = 0.40, *R* squared = 0.02							
***HEI = vmPFC + aVS***							
0	Intercept	58.5	1.88	54.77, 62.23		31.19	<0.001
	vmPFC	0.16	0.21	−0.25, 0.56	0.09	0.76	0.45
	aVS	−0.42	0.29	−0.99, 0.14	−0.18	−1.48	0.14
Model summary: *F*_(2, 89)_ = 1.10, *p* = 0.34, *R* squared = 0.024							
**B) RELATIONSHIP WITH SELF-REPORTED VALUE (BIDS) BY FOOD TYPE**							
***BMI = Healthy + Unhealthy + Interaction***							
0	Intercept	30.43	1.43	27.60, 33.27		21.31	<0.001
	Healthy	−0.21	1.4	−2.99, 2.58	−0.016	−0.15	0.88
	Unhealthy	1.82	1.26	−0.69, 4.32	0.16	1.44	0.15
Model summary: *F*_(2, 90)_ = 1.07, *p* = 0.35, *R* squared = 0.023							
1	Intercept	29.53	3.25	23.07, 35.98		9.09	<0.001
	Healthy	0.71	3.26	−5.78, 7.19	0.05	0.22	0.83
	Unhealthy	3.23	4.73	−6.15, 12.62	0.28	0.69	0.5
	Interaction	−1.38	4.42	−10.15, 7.40	−0.16	−0.31	0.76
Model summary: *F*_(3, 89)_ = 0.74, *p* = 0.53, *R* squared = 0.024							
*R* squared change = 0.001, *p* = 0.76							
***HEI = Healthy + Unhealthy + Interaction***							
0	Intercept	59.62	4.61	50.45, 68.78		12.92	<0.001
	Healthy	6.89	4.52	−2.09, 15.87	0.16	1.53	0.13
	Unhealthy	−13.48	4.08	−21.60, −5.37	−0.34	−3.30	0.001
Model summary: *F*_(2, 89)_ = 5.68, *p* = 0.005, *R* squared = 0.113							
1	Intercept	57.47	10.49	36.62, 78.31		5.48	<0.001
	Healthy	9.07	10.54	−11.87, 30.01	0.21	0.86	0.39
	Unhealthy	−10.12	15.27	−40.46, 20.22	−0.26	−0.66	0.51
	Interaction	−3.26	14.26	−31.60, 25.08	−0.11	−0.23	0.82
Model summary: *F*_(3, 88)_ =3.76, *p* = 0.014, *R* squared = 0.114							
*R* squared change = 0.001, *p* = 0.82							
**C) RELATIONSHIP WITH NEUROCOGNITIVE VALUATION BY FOOD TYPE**							
***BMI = Healthy + Unhealthy + Interaction***							
0	Intercept	30.86	0.57	29.73, 31.99		54.39	<0.001
	Healthy	0.04	0.04	−0.05, 0.13	0.1	0.91	0.37
	Unhealthy	0.06	0.03	−0.02, 0.14	0.16	1.53	0.13
Model summary: *F*_(2, 90)_ = 1.37, *p* = 0.26, *R* squared = 0.03							
1	Intercept	31.14	0.62	29.90, 32.38		49.99	<0.001
	Healthy	0.01	0.05	−0.09, 0.11	0.03	0.24	0.81
	Unhealthy	0.03	0.05	−0.06, 0.13	0.09	0.68	0.5
	Interaction	0.004	0.003	−0.003, 0.01	0.14	1.08	0.28
Model summary: *F*_(3, 89)_ = 1.30, *p* = 0.28, *R* squared = 0.04							
R squared change = 0.013, *p* = 0.28							
***HEI = Healthy + Unhealthy + Interaction***							
0	Intercept	57.17	1.96	54.28, 61.06		29.22	<0.001
	Healthy	0.03	0.15	−0.26, 0.33	0.02	0.22	0.83
	Unhealthy	0.02	0.14	−0.25, 0.29	0.02	0.14	0.89
Model summary: *F*_(2, 89)_ = 0.03, *p* = 0.97, *R* squared = 0.001							
1	Intercept	54.78	2.07	50.66, 58.90		26.43	<0.001
	Healthy	0.27	0.17	−0.06, 0.58	0.19	1.6	0.11
	Unhealthy	0.26	0.16	−0.05, 0.57	0.21	1.65	0.1
	Interaction	−0.032	0.01	−0.06, −0.009	−0.37	−2.78	0.007
Model summary: *F*_(3, 88)_ = 2.60, *p* = 0.057, *R* squared = 0.081							
R squared change = 0.285, *p* = 0.007							

#### Whole-Brain Analyses

The whole-brain search for regions linearly related to subjective value confirmed the involvement of our *a priori* ROIs, and additionally revealed an extended network of brain regions that showed a positive linear relationship with self-reported value. These included a cluster traversing much of the cingulate cortex, large swaths of medial and lateral PFC, dorsal attention network regions, the ventral visual stream, and dorsal and ventral portions of the striatum. We also observed negative relationships between subjective value and the BOLD response in the left visual cortex and bilateral somatosensory cortices (**Figure 4A**, Table 2A). Finally, a whole-brain search of brain areas showing a relationship with BMI and HEI revealed no significant clusters.

**Table 2:** Clusters from whole-brain results for a) overall valuation, and b) reactivity by food type.

				**MNI Coordinates (center)**		
**Region**	**Side**	**Cluster *k***	**Peak *t***	**x**	**y**	**z**
**A) ALL FOODS VALUATION**, ***p*** **< 0.001, k = 119**						
**Positive relationship w/self-reported value**						
Postcentral gyrus (peak)^*^	L	29,602	8.92	−42	−24	58
Inferior parietal lobule-angular gyrus	R	1,950	6.11	43.2	−50.6	49.4
Inferior temporal gyrus	L	1,175	9.25	−59.8	−49.2	−13.2
Cerebellum	L	1,070	5.57	−38.3	−66	−41.5
Inferior temporal gyrus	R	921	6.66	59.5	−46.1	−15.2
Middle frontal gyrus	R	659	7.58	45.8	42.6	14.7
Inferior frontal gyrus-opercularis	R	449	5.89	51.1	10.1	20.3
Superior frontal gyrus	R	402	4.31	29.9	15.2	57.3
Anterior insula	R	135	6.14	36.5	20.1	−0.2
**Negative relationship w/self-reported value**						
Lingual gyrus, visual cortex	L	1,426	−10.58	−10.9	−85.5	−8.5
Precentral gyrus	R	505	−6.91	51.7	−11.2	46.8
Postcentral gyrus	L	501	−12.96	−48.2	−16.8	48.3
Postcentral gyrus	R	162	−4.33	24.8	−37.2	67.5
**B) FOOD-CUE REACTIVITY**, ***p*** **< 0.001, k = 119**						
**Healthy > Unhealthy**						
Superior-inferior parietal lobule	L	2,681	5.81	−32.7	−65.7	46.3
Dorsolateral prefrontal cortex	L	2,535	5.35	−52	12	30
Inferior temporal gyrus	L	1,256	7.76	−55.7	−57.5	−10
Angular gyrus	R	1,239	3.84	34.3	−63	44.4
Inferior temporal gyrus	R	1,168	7.04	54.7	−56.8	−12.7
Inferior frontal gyrus-opercularis	R	440	5.01	45.6	9.7	27.9
Supplementary motor area	L	427	5.05	−1	18.2	51.7
Inferior frontal gyrus-triangularis	R	281	4.68	47.2	36.3	15.1
Cerebellum	R	246	5.97	35.9	−70.3	−51.7
Precuneus	L	205	5.75	−5.5	−55.1	9.8
Cerebellum	R	121	5.29	34.3	−64.8	−29.6
**Unhealthy > Healthy**						
Calcarine gyrus, visual cortex	L	8,119	−16	−4.1	−90.4	−4.4
Middle cingulate	R	289	−4.95	2.1	−20.4	42
Postcentral gyrus	L	267	−6.09	−46.9	−17.1	47.3
Middle frontal gyrus	R	215	−4.24	26.4	56	23.2
Temporal pole	R	150	−4.44	58	14	−4
Middle temporal gyrus	R	135	−3.71	63.6	−23.2	−6.7

* *One contiguous cluster that spans most of the left parietal lobe, most of the cingulate gyrus, much of the mPFC, left dlPFC, left AI, most of the striatum, parts of the hippocampus, and brain stem*.

### Valuation by Food Type

#### Behavioral

**Figure 2:**
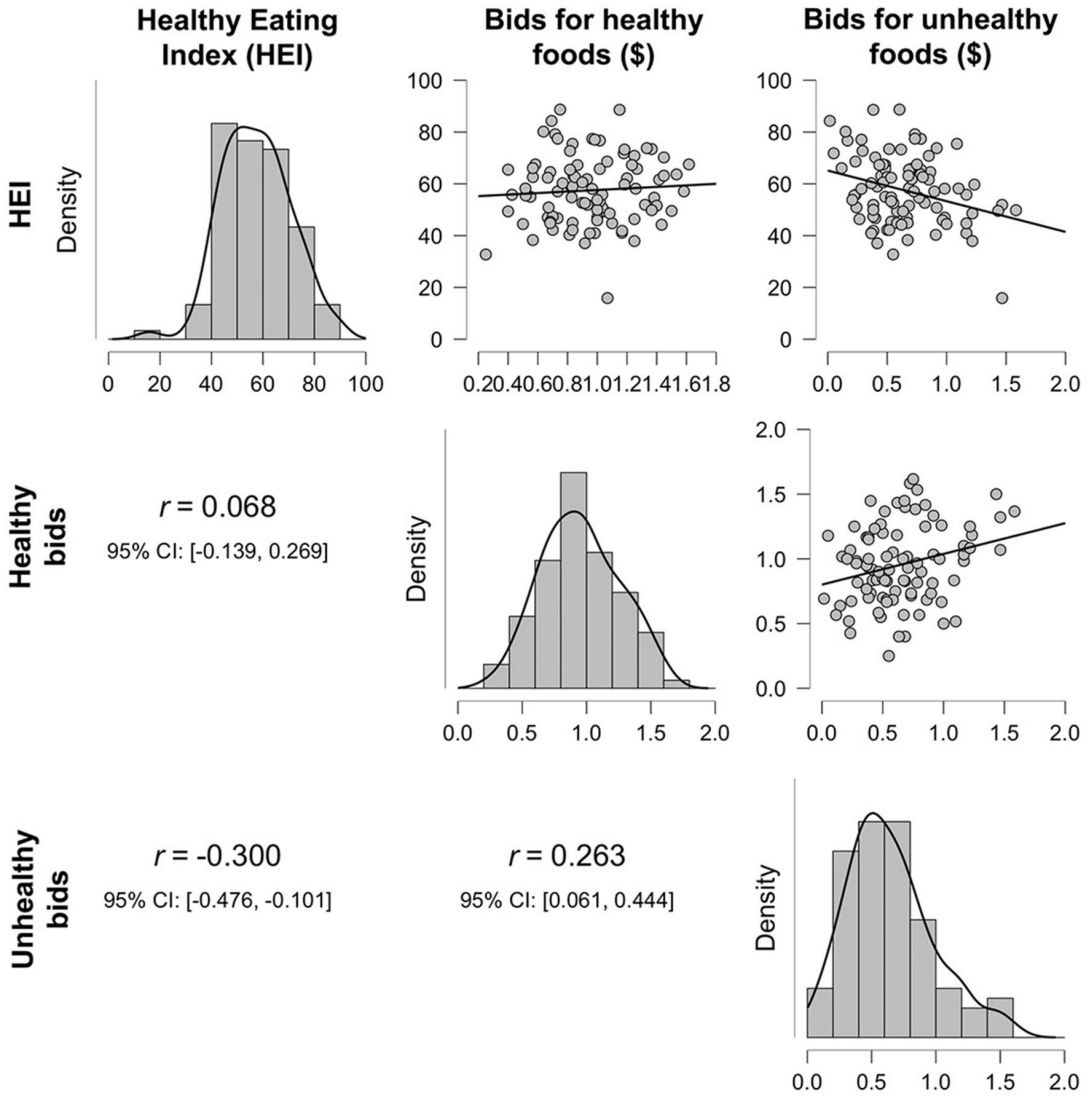
Density plots and correlations between the healthy eating index (HEI), average bid amounts for healthy foods, and average bid amounts for unhealthy foods.

Behaviorally, we did not find a significant relationship when regressing BMI on the averaged bid value for healthy and unhealthy foods, nor when the interaction was included, *p* > 0.35. Bayes Factor suggested strong evidence in favor of the null hypothesis for the model including the interaction term, BF_10_ = 0.07, indicating that it is quite likely that there truly is no relationship. When regressing HEI on the averaged bid values for healthy and unhealthy foods, we did find a significant relationship, *F*_(2, 89)_ = 5.68, *p* = 0.005, *R*^2^ = 0.11. Partial model coefficients indicate that this effect was driven by a negative relationship between HEI and unhealthy food bids, B = −13.48, SE = 4.08, 95% CI [−21.60, −5.37], *t* = −3.30, *p* = 0.001 (Figure 2; Table 1B), suggesting that participants who bid higher for unhealthy foods ate less healthily in the real world. The inclusion of the interaction term in the second step of this model did not significantly add to the variance explained, *p* = 0.82, further suggesting that this effect is specific just to bids for unhealthy foods.

#### ROI Analyses

**Figure 3:**
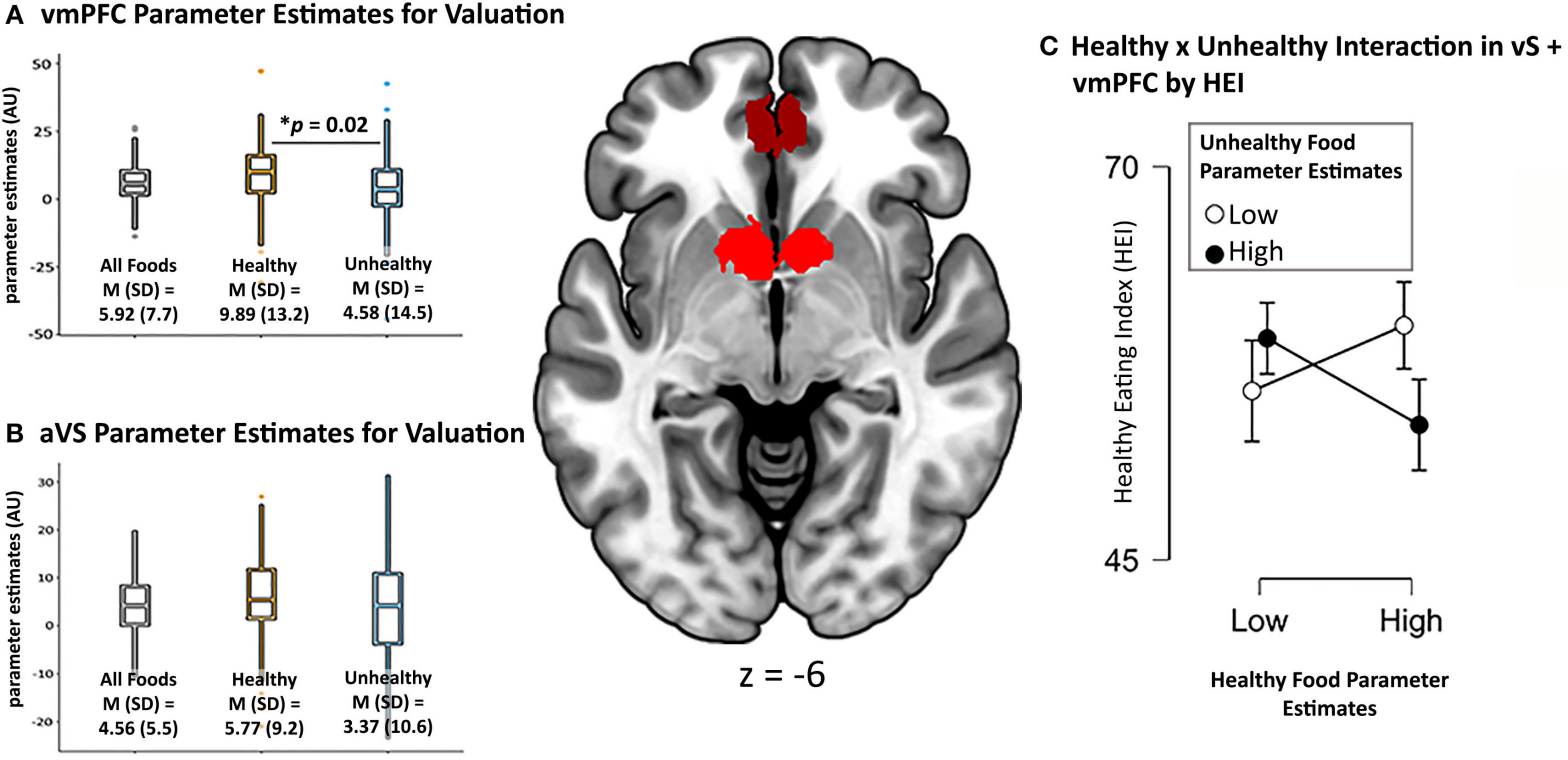
vmPFC and aVS ROIs used in analysis pictured in center image. Parameter estimates in arbitrary units (AU) of overall valuation of all foods, healthy foods, and unhealthy foods from **(A)** vmPFC and **(B)** aVS. The interaction between valuation for healthy and unhealthy foods, and its relation to healthy eating **(C)** plotted using median split of the vmPFC + VS combined parameter estimates of healthy and unhealthy foods (even though data are continuous).

When comparing the parameter estimates for valuation of healthy > unhealthy foods within each of our *a priori* ROIs, the vmPFC showed a stronger relationship (i.e., larger beta value) for healthy compared to unhealthy foods, M_diff_ = 5.31, SD = 21.53, 95% CI [0.88, 9.75], *t*(92) = 2.38, *p* = 0.02 (Figure 3A), while there was no such difference in the aVS, *p* = 0.12 (Figure 3B). This suggests that, while the aVS tracked value for both food categories relatively equally, the coupling of self-reported value and corresponding BOLD response in the vmPFC was stronger for healthy foods, possibly due to the integration of non-desire related value inputs.

We did not find a significant relationship when regressing BMI on our neurocognitive measures of valuation for healthy and unhealthy foods, even when including the interaction term, *p* >0.26. Bayes Factor of the model including the interaction term suggested moderate evidence in favor of the null hypothesis, BF_10_ = 0.13. When regressing HEI on these measures, the model including the interaction term to predict HEI showed a trending effect, *F*_(3, 88)_ = 2.60, *p* = 0.057, *R*^2^ = 0.08. Partial model coefficients indicated that this effect was driven by the interaction term, B = −0.03, SE = 0.01, 95% CI [−0.06, −0.01], *t* = −2.78, *p* = 0.007 (Table 1C). Using median split to probe the direction of the interaction revealed that participants who exhibited high parameter estimates (i.e., stronger coupling between self-reported value and corresponding BOLD response) for healthy foods *and* low parameter estimates for unhealthy foods reported eating healthier in the real world than participants who had high parameter estimates for both food categories, while participants exhibiting low parameter estimates for healthy foods did not show differences in their healthy eating regardless (Figure 3C). Follow-up, exploratory regression models that separated out the ROIs suggested that valuation of the food types in the vmPFC largely contributed to this effect, *F*_(3, 88)_ = 2.20, *p* = 0.094, *R*^2^ = 0.07, again driven by the interaction term, B = −0.01, SE = 0.01, 95% CI [−0.02, −0.002], *t* = −2.29, *p* = 0.024, while the regression model of just aVS showed no such effect, *p* =0.7. Together, these results suggest that, rather than either food category alone, it is the neurocognitive valuation of both healthy *and* unhealthy foods that relates to real world eating behavior.

#### Whole-Brain Analyses

The whole-brain contrast between the valuation of healthy vs. unhealthy foods revealed no significant clusters at our predetermined threshold of *p* < 0.001, k = 119. However, when relaxing the threshold to an uncorrected *p* < 0.005, k = 300, we found clusters in the vmPFC and pre-SMA, which provide converging evidence with our ROI analysis that the vmPFC shows a stronger relationship for healthy vs. unhealthy foods (Figure 3A, green). A whole-brain search looking for relationships between this contrast and BMI revealed no significant clusters, nor did the whole-brain search for HEI.

### Replication Attempt

**Figure 4:**
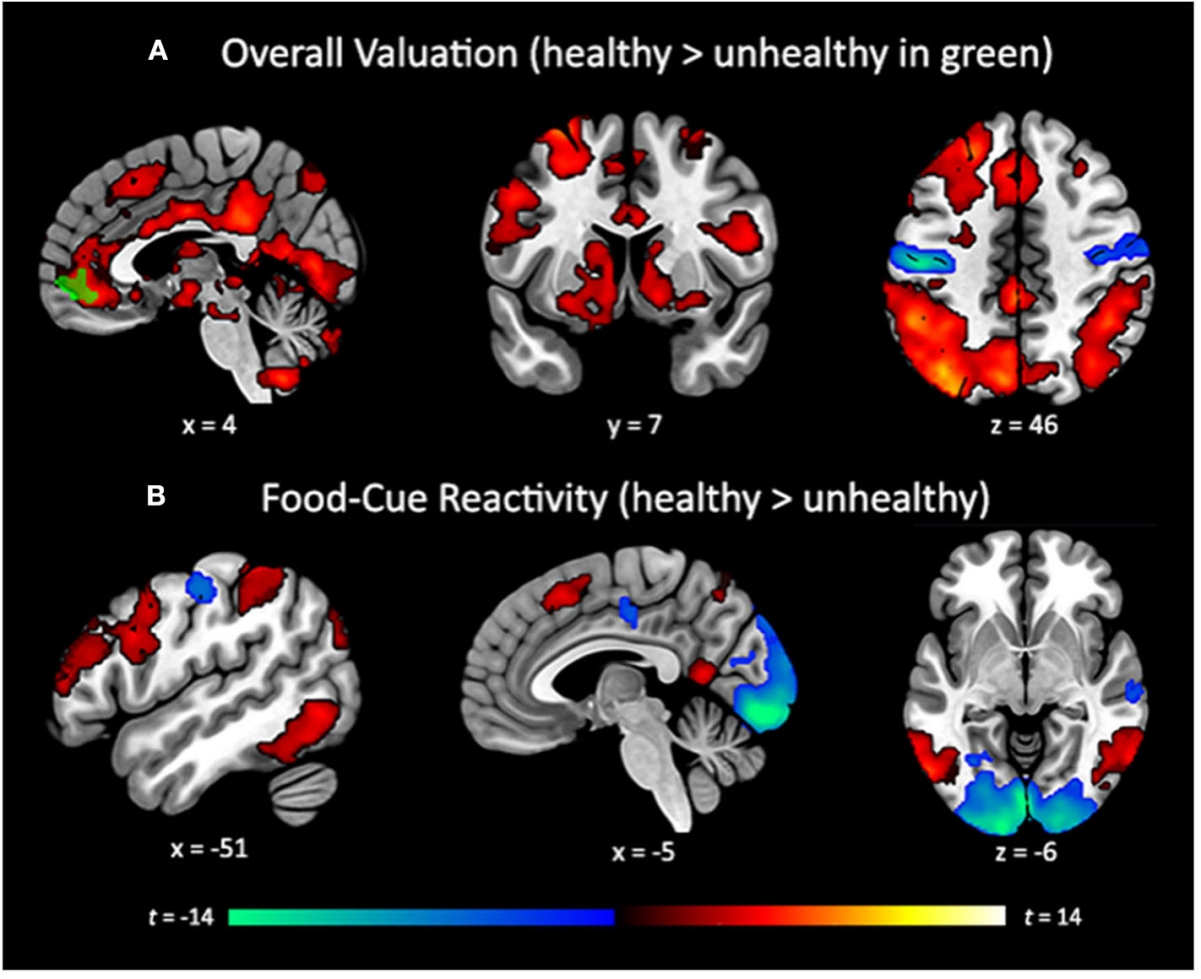
**(A)** Whole-brain search of subjective valuation for all foods with positive (hot colors) and negative (cold colors) coupling between bid value and corresponding BOLD response (cluster corrected *p* < 0.05). vmPFC cluster (green) showing greater coupling for healthy than unhealthy foods (*p* < 0.005, k = 300, uncorrected). **(B)** Whole-brain contrast between reactivity for healthy (hot colors) and unhealthy foods (cold colors) not accounting for bid value (cluster corrected *p* < 0.05). Cluster correction of *p* < 0.001, k = 119 to achieve a whole-brain familywise error rate of α = 0.05.

In our attempt to provide a conceptual replication of Verdejo-Román and colleagues (2017) who contrasted reactivity to palatable > unpalatable foods, we contrasted reactivity to unhealthy > healthy foods, but we did not find similar results. The unhealthy > healthy contrast showed considerable activation in the visual cortex, posterior to mid-cingulate, central sulcus, and anterior portion of the right middle frontal gyrus. The healthy > unhealthy contrast revealed extended activation traversing the dorsal attention network into bilateral dlPFC and IFG, bilateral regions of the dorsal and ventral visual streams, supplementary motor area, and precuneus (Figure 4B; Table 2B). While lateral prefrontal activation was reported by Verdejo-Román et al. (2017), our result is in the opposite direction of what would qualify as a conceptual replication, such that they reported this cluster for the unpalatable > palatable contrast, while we found this for the healthy > unhealthy contrast. We also were unable to replicate their findings when comparing obese > overweight individuals for this contrast, such that we found no significant clusters, even when using very liberal thresholds. Moreover, we conducted an exploratory whole-brain search of BMI on the healthy > unhealthy contrast, and still found no significant clusters.

## Discussion

The current study provides the first examination of how subjective valuation of food items relates to BMI and healthy eating in a sample of early middle-aged, higher BMI adults motivated to eat more healthily. Our findings suggest normative functioning in the brain's valuation system during food-based decision making, such that there were robust linear relationships between self-reported value of food items and the corresponding BOLD response in the vmPFC, aVS, bilateral AI, and the ACC when making decisions about snack food items. Further, we found moderate evidence in favor of a null relationship between our estimates of overall valuation with both BMI and HEI. Together, these findings add to the health neuroscience literature by suggesting that higher BMI individuals do not display biases in the neurocognitive substrates of subjective valuation, and that the general functioning of this system may not relate to BMI or healthy eating. When examining differences in the self-reported valuation of healthy and unhealthy foods, our results indicate that self-reported valuation of unhealthy foods is inversely related to real world healthy eating, but we found no relationship for the valuation of either food type with BMI. This finding echoes prior work demonstrating a relationship between unhealthy eating behavior and reactivity to unhealthy food cues (Tetley et al., 2009), and suggests that valuation may be an important factor underlying this relationship (Cosme and Lopez, 2020). Moreover, this highlights the importance of regulation strategies that target the devaluation of unhealthy foods, whether by reappraisal or other down-regulation strategies (Giuliani et al., 2014).

Interestingly, despite reporting higher overall value for healthy compared to unhealthy foods (Cosme et al., 2019), we found no relationship with the self-reported valuation of healthy foods and real-world healthy eating behavior or BMI. One possibility is that the motivation of people in this sample, who volunteered for a healthy eating study, to eat more healthily might have contributed to higher self-reported value for healthy foods, whereas the self-reported value of unhealthy foods tracked with an elevated craving for unhealthy foods that more strongly predict real-world eating behavior when considering only self-report data. This interpretation is in line with the value-based models of health-oriented behavior that point to the vmPFC as a hub where various motivational factors are integrated (Berkman, 2018), and is partially supported by our neuroimaging findings that demonstrate a stronger coupling between self-reported value and BOLD response in the vmPFC for healthy compared to unhealthy foods overall. Importantly, the interaction between the valuation process for healthy and unhealthy foods in this region is related to healthy eating, such that the participants who show higher neurocognitive coupling for healthy foods *and* lower coupling for unhealthy foods exhibited the healthiest eating. However, these findings need to be validated by future work given that our full model was only marginally significant. Nonetheless, these findings suggest that the vmPFC may integrate health motivations during the subjective valuation of healthy foods, and the interaction between valuation of healthy and unhealthy foods predicts healthy eating. Future research should also obtain quantitative measures of health motivation to better test the value integration model.

We were unable to replicate the findings of Verdejo-Román et al. (2017), which showed greater activation in subcortical reward circuitry, and medial and lateral portions of the prefrontal cortex when contrasting palatable vs. unpalatable foods. The motivation for this replication attempt relied on the assumption that palatability is inversely related to healthiness, such that unhealthy foods are more palatable (e.g., pizza), while healthy foods are less palatable (e.g., whole-grain oats). However, it is not necessarily the case that food healthiness relates to its palatability. Indeed, many of the healthy food items used as stimuli in the willingness-to-pay task were pictured in appealing ways (e.g., a bunch of fresh grapes), while some of the unhealthy items were less so (e.g., a bag of chips). A better approach would have been to re-categorize our stimuli based on palatability ratings rather than using our existing categories of healthy and unhealthy foods. Another promising direction for future research would be to examine the nutritional content of food stimuli as it relates to food-cue reactivity and the subjective valuation process. Recent work has demonstrated that different areas of the prefrontal cortex can track caloric density (Tang et al., 2014) and represent macro-nutrients (Suzuki et al., 2017), while the combination of fat and carbohydrates have supra-additive effects on striatal response (DiFeliceantonio et al., 2018). By quantifying the various dimensions of food stimuli that are encoded by the brain, we can gain a more complete picture of the neural mechanisms underlying weight and eating behavior. Thus, while we were unable to provide converging evidence for the neural systems that are sensitive to palatability as reported by Verdejo-Román and colleagues (2017), it is likely due to a mismatch the dimensions of health and palatability.

Our healthy food-cue reactivity contrast did demonstrate greater activity in lateral prefrontal, dorsal attention, and higher order visual processing regions, while unhealthy foods elicited greater activity in primary visual and somatosensory regions. These findings suggest that healthy foods engaged higher order, top-down processing networks, while unhealthy foods caused reactivity in bottom-up perceptual systems. The engagement of top-down processing may be due to the fact that our sample was motivated to eat more healthily, and therefore engaged more effortful deliberation when making decisions about healthy foods. This interpretation is line with studies showing the engagement of these regions during regulatory processes (see Buhle et al., 2014 for meta-analysis). The pattern of activation for unhealthy foods mirrors the results from traditional studies of food-cue reactivity (Murdaugh et al., 2012).

It is noteworthy that, throughout all our analyses, we found no relationships between brain activity and BMI, which is similar to results from other experiments in this sample (Giuliani et al., 2020). While researchers have argued for better measures of obesity as it relates to health (Ahima and Lazar, 2013), there are a number of reasons we do not see a relationship between BMI and brain response in the valuation system as reported in other studies (e.g., Petit et al., 2016). First, this may be due entirely to the fact that we were studying a sample that desired to eat more healthily, thus the self-reported and neural responses we observed might not be comparable to what has been reported in prior literature. This interpretation is supported by research showing that when individuals attend to the health aspects of food, it improves dietary choice and modulates the neural responses associated with the valuation process (Hare et al., 2011). Thus, it is likely that people in our sample—who were motivated to eat more healthily and who enrolled in a healthy eating study—were more attentive to food healthiness. However, there are no studies to our knowledge that have examined healthy eating motivation as mediator between BMI and brain response to food cues, so this interpretation requires further investigation. Another major shortcoming that may have contributed to this null result is the fact that the current study did not include healthy-weight controls. Thus, the restricted range of BMI we investigated may have reduced the variability needed to uncover the relationship with BMI reported in previous literature. Relatedly, it is possible that there are categorical differences between healthy-weight and higher BMI individuals in how they process food stimuli, as suggested by the food addiction model (Volkow et al., 2013b), which may further obscure a relationship with weight. Finally, it is possible that these null results were due to measurement error, since we measured participant weight in the lab. Because we did not have a clinical setting to provide lightweight scrubs to all participants, combined with the fact that these data were collected on a rolling basis over the course of 2 years, differences in clothing weight across the seasons may have contributed unsystematic noise to our measurement of weight, and, consequently, BMI.

Taken together, the current study adds to the health neuroscience literature by demonstrating both significant *and* null findings across a set of well-motivated, pre-registered analyses. We provide evidence that higher BMI individuals exhibit normative coupling between self-reported value and corresponding BOLD response. These individuals also show meaningful relations between value-related brain activation and eating patterns. These results add to the knowledge base on how people with higher BMI process and relate to food stimuli. The null results are equally informative to the literature in this area. Interestingly, the neurocognitive measures of food valuation do not relate to BMI or healthy eating behaviors. The lack of a relation provides a possible explanation for why such a relationship has not been reported in prior work. Moreover, we demonstrated a potential mechanism that contributes to real-world eating behavior—the valuation of healthy and unhealthy foods. These findings direct future research toward further examinations of motivational factors and food-cue information that are integrated in the vmPFC during food-based decision making, and suggest that interventions should target both the up-valuation of healthy foods and down-valuation of unhealthy foods, rather than focusing on one or the other exclusively.

## Data Availability Statement

The datasets generated for this study can be found in online repositories. The names of the repository/repositories and accession number(s) can be found at: https://neurovault.org/collections/GDNZHILR/.

## Ethics Statement

The studies involving human participants were reviewed and approved by Institutional Review Board at the University of Oregon. The patients/participants provided their written informed consent to participate in this study.

## Author Contributions

NG and EB designed the study. JM, BD, and NG collected the data. JM and DC analyzed the data. JM wrote the manuscript and created the figures. NG, DC, BD, and EB provided edits. All authors contributed to the article and approved the submitted version.

## Conflict of Interest

EB is manager of Berkman Consultants, a boutique consulting firm specializing in goals, motivation, and behavior change. The remaining authors declare that the research was conducted in the absence of any commercial or financial relationships that could be construed as a potential conflict of interest.
